# Methods for monitoring and measurement of protein translation in time and space

**DOI:** 10.1039/c7mb00476a

**Published:** 2017-10-12

**Authors:** Maria Dermit, Martin Dodel, Faraz K. Mardakheh

**Affiliations:** a Centre for Molecular Oncology , Barts Cancer Institute , Queen Mary University of London , John Vane Science Centre , Charterhouse Square , London EC1M 6BQ , UK . Email: f.mardakheh@qmul.ac.uk

## Abstract

Regulation of protein translation constitutes a crucial step in control of gene expression. In comparison to transcriptional regulation, however, translational control has remained a significantly under-studied layer of gene expression. This trend is now beginning to shift thanks to recent advances in next-generation sequencing, proteomics, and microscopy based methodologies which allow accurate monitoring of protein translation rates, from single target messenger RNA molecules to genome-wide scale studies. In this review, we summarize these recent advances, and discuss how they are enabling researchers to study translational regulation in a wide variety of *in vitro* and *in vivo* biological systems, with unprecedented depth and spatiotemporal resolution.

## Abstract 2

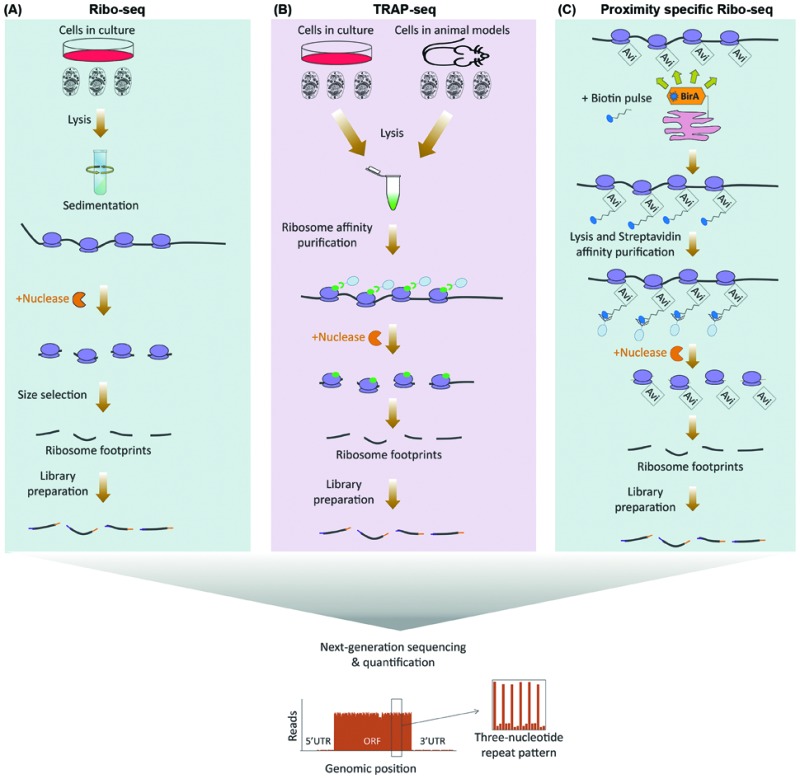
Regulation of protein translation constitutes a crucial step in control of gene expression. Here we review recent methods for system-wide monitoring and measurement of protein translation.

## Introduction

The central dogma of molecular biology states that genetic information flows sequentially from DNA, *via* messenger RNA (mRNA), to proteins, the often final functional products of gene expression.^1^ As a result of decades of intense research in both prokaryotes and eukaryotes, it is mechanistically well understood how DNA is transcribed and processed into mRNA, and how mRNA is translated into proteins. However, a key issue remaining is how the rates of information flow – from genes to proteins – are regulated, and how the protein levels are defined inside the cell at any given time. As transcription initiates the cascade of genetic information flow, it had long been assumed to be the defining step in regulation of gene expression. Thanks to a variety of global transcriptome analysis methods such as DNA micro-array chip hybridization (micro-array)^2^ and RNA-sequencing (RNA-seq),^3^ much has been revealed regarding regulation of gene expression at the mRNA level. In addition, Chromatin Immunoprecipitation (ChIP) followed by micro-array chip hybridization (ChIP-chip)^4^ or next-generation sequencing (ChIP-seq)^5^ methods have enabled analysis of transcription factor-DNA associations on genome-wide scales. This has brought about system level understanding of transcriptional networks and their regulation in a wide variety of biological systems, and in response to various physiological or pathological modulations. However, little is known about how gene expression at the level of translation is regulated. Ironically, multitude of studies using quantitative proteomics in conjugation with transcriptomics have highlighted that globally little correlation exists between mRNA and protein levels in various biological systems,^6^–^11^ although individual protein/mRNA ratio levels seem to be conserved.^12^,^13^ This suggests that the bulk of gene expression regulation must occur post-transcriptionally. Crucially, with the advent of methods that allow measurement of protein translation rates on a global scale, it has become apparent that translational control seems to be the defining step in determining the steady-state levels of most cellular proteins.^14^,^15^ Consequently, the interest in studying the impacts of translational regulation has greatly surged in recent years. This has been matched by development of a plethora of diverse methodologies which allow assessment of protein translation *in vitro* and *in vivo*, and from the scale of individual target mRNAs all the way to genome-wide studies. In this review, we compare and contrast these methods and their applications, and discuss how they can be used to reveal the dynamics of translational regulation (Table 1). We also discuss how these methods can be utilized to spatially resolve the sites of protein translation inside the cell, and implication of such spatial information on our understanding of the role and significance of localized translation in cell biology.

**Table 1:** List of methods for analysis of translation in time and space

	Method	Advantages	Disadvantages	Ref.
Next-generation sequencing	Polysome profiling	Reproducible; quantitative; high depth of analysis; gives an instantaneous snapshot of the translatome (high temporal resolution).	Contamination by co-sedimented RNPs can be an issue; does not reveal the exact ORF sites in an mRNA; more association of an mRNA to ribosomes may not always mean more translation.	16
	Ribo-seq	High depth of analysis; single-nucleotide resolution; allows *de novo* ORF detection; highly quantitative; gives an instantaneous snapshot of the translatome (high temporal resolution).	Costly and time consuming; requires a large amount of starting material; more association of an mRNA to ribosomes may not always mean more translation.	15 and 20
	TRAP-seq	Similar to ribo-seq but can be used for cell-specific *in vivo* analysis of translation.	Similar to ribo-seq, but requires more starting material.	33 and 34
	Proximity-specific ribo-seq	Similar to ribo-seq but can reveal subcellularly localized translation.	Similar to TRAP-seq, but requires even more starting material as only a fraction of total cellular ribosomes are labeled and purified.	36 and 37

Proteomics	p-SILAC	Quantitative; measures nascent proteins; allows analyses from small sample sizes and subcellular compartments.	Low depth; limited temporal resolution due to the need for incorporation of pulsed amino acids into cellular proteins; cannot be readily used *in vivo*.	14, 39 and 41
	BONCAT	Measures nascent proteins; higher depth than p-SILAC due to enrichment of nascent proteins.	Limited temporal resolution due to the need for incorporation of pulsed amino acids into cellular proteins; cannot be readily used *in vivo* without utilizing engineered amino acyl-tRNA synthetases; semi-quantitative.	46
	SORT	Similar to BONCAT but can be used for cell-specific *in vivo* analysis of translation.	Generation of animal models costly and time consuming. Limited temporal resolution due to the need for incorporation of pulsed amino acids into cellular proteins; semi-quantitative.	54
	QuaNCAT	Quantitative like p-SILAC, but at higher depths due to enrichment of nascent proteins; improved temporal resolution in comparison to BONCAT and p-SILAC; measures nascent proteins.	Improved, but still limited temporal resolution due to the need for amino acid pulsing; cannot be readily used *in vivo.*	56
	HILAQ	Quantitative like p-SILAC, but at higher depths; experimental workflow much simpler that QuaNCAT; improved depth and temporal resolution in comparison to QuaNCAT.	Improved, but still limited temporal resolution due to the need for amino acid pulsing; cannot be readily used *in vivo.*	59
	PUNCH-P	High depths of analysis; gives an instantaneous snapshot of the translatome (high temporal resolution); measures nascent proteins.	Time consuming; requires prior lysis and purification of translating ribosomes, thus losing any spatial regulatory influences on translation; requires a large amount of starting material; semi-quantitative.	63
	OPP capture	Improved temporal resolution compared to p-SILAC and BONCAT due to rapid OPP incorporation into cellular proteins; measures nascent proteins; can be used for cell-specific *in vivo* assessment of translation (PhAc-OPP).	Semi-quantitative (as of now).	64 and 65

Live cell imaging	TRICK	Allows live monitoring the first round of translation; single molecule sensitivity; can potentially be used *in vivo*.	Not high throughput; low signal to noise ratio; cannot be used for assessment of translation rates, but only visualizing the pioneer round of translation.	67
	NCT/SINAPS	Allows continuous monitoring of translation dynamics in live cells over time scales of hours; single molecule sensitivity; reveals translation heterogeneity; can potentially be used *in vivo*.	Not high-throughput; background fluorescent accumulation over time can be an issue.	69–72

## Next-generation sequencing based analysis of translation

**Fig. 1:**
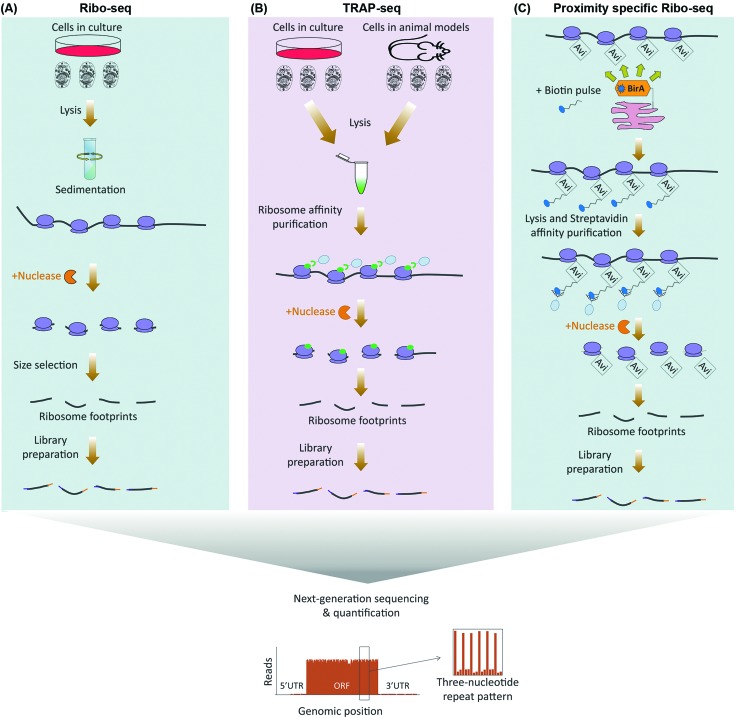
Next-generation sequencing based methods for global analysis of translation. (A) In ribo-seq, ribosome-bound mRNAs are purified by sedimentation following cell lysis. Nuclease treatment is then used to degrade unmasked RNA sections, leaving 20–30 nucleotide-long ribosome protected fragments known as ribosome footprints. The footprints are then subjected to library preparation and next-generation sequencing (below). The read densities can be used to both distinguish individual ORFs, as well as to quantify their translation rates, with a distinctive three-nucleotide periodic footprint pattern that is indicative of ribosome translocation often visible throughout the length of the ORF. (B) TRAP-seq differs from ribo-seq in the way ribosomes are purified. Rather than sedimentation, epitope tagged ribosomal proteins which are either stably expressed in cultured cells or transgenically in a given cell-type of an *in vivo* model, are subjected to immunoprecipitation in order to pulldown the translating ribosomes. Nuclease treatment is then used to degrade unmasked RNA sections, followed by library preparation, and next-generation sequencing of the footprints as before (below). (C) Proximity-specific ribo-seq allows assessment of subcellularly localized translation by tagging ribosomal proteins with a biotin acceptor peptide (Avi), coupled with expression of a subcellularly localized biotin ligase (BirA). A carefully optimized biotin pulse is then applied in order to induce biotinylation of the Avi tagged ribosomes in close proximity of the BirA. Subsequently, cells are lysed and biotinylated ribosomes are affinity purified using streptavidin conjugated beads, followed by nuclease treatment, library preparation, and next-generation sequencing of the ribosome footprints as before (below).

As initiation is often the rate-limiting step in regulation of protein translation, association of mRNAs with translating ribosomes can be used as a proxy for estimation of translation rates (Table 1). Ribosome association with mRNAs can be monitored by purifying polysomes through sedimentation in a sucrose gradient, and assessing relative mRNA enrichment levels in the polysomal fraction by micro-array or next-generation sequencing analysis, a method known as polysome profiling.^16^ Despite its robust methodology, however, polysome profiling suffers from a number of drawbacks. Simply equating the co-sedimentation of an mRNA with polysomes to its translation is rather a crude assumption, as several other large Ribonucleoprotein (RNP) complexes can co-sediment with polysomes.^17^ Moreover, polysome profiling does not take into account the well-known fact that apart from the major Open Reading Frame (ORF), additional ORFs in the 5′ UTR of mRNAs that are known as upstream ORFs (uORF) exist in around half of all cellular transcripts, which can be independently undergoing translation.^18^ ribosome profiling (ribo-seq), mitigates these shortcomings by enabling position sensitive assessment of translation on a genome-wide scale.^15^ In this method, translation is first halted through rapid detergent-based lysis, flash-freezing, or use of ribosome translocation inhibitors such as cycloheximide. The mRNA–ribosome complexes are then purified by sedimentation and subjected to nuclease treatment, leaving 20–30 nucleotide long ribosome protected mRNA fragments known as ribosome foot-prints. These footprints can be accurately identified and quantified with single nucleotide resolution, using RNA-seq^19^ (Fig. 1A).

The single nucleotide resolution of ribo-seq enables the exact sequence identity of all translating ORFs to be systematically revealed on a genome-wide scale. Consequently, ribosome profiling studies have been able to reveal widespread translation occurring outside of canonical ORFs. These include translation from alternative initiation sites, use of non-AUG start codons, overlapping ORFs, and stop codon bypasses.^20^–^24^ In addition, widespread translation can also be mapped to uORFs, as well as other short ORFs (sORF) which reside in RNAs that were previously thought to be non-coding.^20^,^23^,^25^–^28^ Apart from revealing non-canonical ORFs, a key feature of ribo-seq is its ability to capture an instantaneous snapshot of the translatome. This, combined with the extreme sensitivity and the broad dynamic range that can be achieved with next-generation sequencing, renders ribo-seq an ideal method for sensitive in-depth quantification of temporal changes in the translatome.^29^–^32^ In addition, as an alternative to sedimentation, translating ribosomes can be purified by immunoprecipitation of epitope tagged ribosomal proteins, a method known as translating ribosome affinity purification-sequencing (TRAP-seq).^33^ Such epitope tagged ribosomal proteins can be transgenically expressed in specific cell-types of a given model organism, thus enabling ribosome profiling to be applied for monitoring of cell-specific translation, *in vivo*^34^ (Fig. 1B).

Ribo-seq can also be adapted for spatial analysis of translation. This can either be done by using subcellular fractionation prior to purifying ribosomes,^35^ or through use of proximity-specific ribosome profiling.^36^,^37^ In the latter, rather than using an epitope tag, ribosomal proteins are tagged with a biotin acceptor peptide (Avi Tag). In parallel, a biotin ligase (BirA) is targeted to a specific subcellular compartment. A carefully optimized biotin pulse can then result in specific biotinylation of ribosomes in the vicinity of localized BirA. Labeled ribosomes are then purified *via* streptavidin conjugated resins for ribo-seq analysis^36^,^37^ (Fig. 1C). Proximity-specific ribosome profiling is particularly useful for assessment of localized translation in subcellular compartments which cannot be separated *via* available cell fractionation methods, or in case available fractionation procedures perturb the subcellular distribution of ribosomes that are associated with a given compartment.

Despite its great advantages, however, ribo-seq suffers from a number of drawbacks. A key assumption in ribo-seq analysis is the uniformity of translation elongation rates amongst all cellular mRNAs.^15^ While this is thought to be true in most cases, some RNA Binding Proteins (RBPs) such as FMRP have been shown to regulate the translation of their target mRNAs by stalling the elongating ribosomes throughout the full lengths of the ORF.^38^ Such regulations at the level of elongation could be misinterpreted when using ribo-seq. Another major limitation of ribo-seq is the needed amounts of starting material. This is due to the fact that at any given time point, only a fraction of total cellular mRNAs tends to be associated with ribosomes. Furthermore, as with any biochemical purification step, efficient separation of ribosomes by sedimentation or TRAP depends on ample amounts of input material. A final drawback is the large number of steps in the sample preparation and analysis pipeline, which makes ribo-seq experiments costly, time-consuming, and prone to potential experimental artefacts.

## Proteomics based analysis of translation

**Fig. 2:**
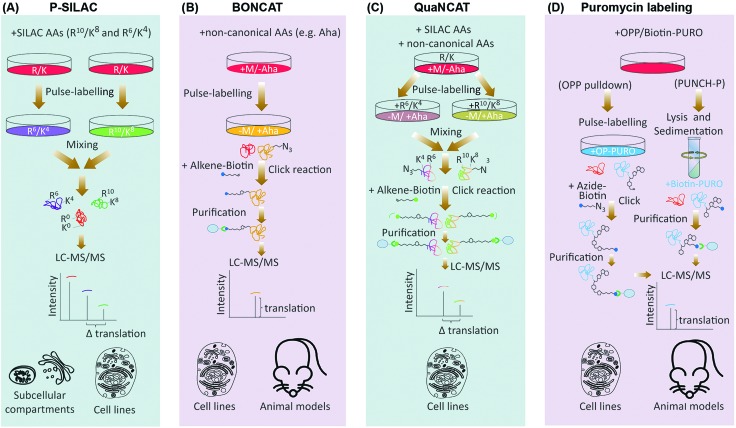
Proteomics based methods for global analysis of translation. (A) In p-SILAC, the differences in protein synthesis rates are directly quantified by LC-MS/MS, through comparing the intensity ratios of SILAC pulsed labeled (K^4^/R^6^*vs.* K^8^/R^10^) nascent proteins. Old, un-labeled (K^0^/R^0^) proteins are not taken into account during the data analysis. (B) In BONCAT, pulse-labeling is done by non-canonical amino acids such as the methionine analogue azidohomoalanine (Aha), which carries an active azide (N_3_) group. Using Click chemistry, pulse-labeled proteins can be covalently attached to an enrichment tag such as biotin, purified using streptavidin conjugated beads, before identification by LC-MS/MS. (C) QuaNCAT combines the principles of p-SILAC and BONCAT to metabolically label nascent proteins with both non-canonical and SILAC amino acids. QuaNCAT allows both enrichment of nascent proteins *via* non-canonical amino acid labeling, as well as accurate relative quantification through SILAC labeling. (D) Puromycin (PURO) based labeling methods use variants of the antibiotic puromycin for labeling and purification of nascent proteins. In PUNCH-P, cells are lysed and the translating ribosomes are separated by sedimentation, before cell-free labeling of the newly synthesized proteins, using a Biotin-PURO conjugate. Alternatively, cells can be pulsed by an alkyne–puromycin conjugate called OPP, which unlike Biotin-PURO is cell permeable, followed by lysis and Click conjugation to biotin. In both methods, labeled nascent proteins are then purified using streptavidin conjugated beads, and identified by LC-MS/MS. AA: amino acids; M: methionine.

In contrast to ribo-seq which evaluates the association of mRNAs with ribosomes as a proxy for protein synthesis, proteomics based methods which have been developed for assessment of translation do so by direct identification and quantification of nascent proteins. This is achieved by labeling nascent proteins through pulses of amino acid isotopologues, non-canonical amino acids, or specific chemical conjugates, followed by purification and quantification of the pulse-labeled proteins *via* mass spectrometry (Table 1). Pulsed-SILAC (p-SILAC)^39^ is one such method, based on Stable Isotope Labeling of Amino acids in Culture (SILAC).^40^ p-SILAC utilizes pulse treatments of stable isotopologues of arginine (Arg) and lysine (Lys), in order to metabolically label newly synthesized proteins at time points defined by the pulse (Fig. 2A). Initially, cells are cultured in presence of unlabeled ‘light’ Arg and Lys. Culture media are then changed to media containing ‘medium’ (Arg6 and Lys4) or ‘heavy’ (Arg10 and Lys8) labeled SILAC amino acids for the duration of the pulse. After samples are harvested, mixed, and processed, peptides are analyzed by Liquid Chromatography coupled with tandem Mass Spectrometry (LC-MS/MS), and newly synthesized proteins containing labeled amino acids are identified and quantified^39^ (Fig. 2A). This approach has been fundamental in determining the translation rates of cellular proteins in mammalian cell cultures, and systematically assessing the contribution of protein translation towards determining the steady-state levels of cellular proteins.^14^ Moreover, as p-SILAC requires minimal sample processing and does not depend on any biochemical purification step after lysis, it enables assessment of protein translation rates from relatively small sample sizes. This has allowed combining p-SILAC with subcellular fractionation in order to determine the localized translation rates of cellular proteins from even small subcellular fractions.^41^ However, a significant shortcoming of p-SILAC is its relative low coverage due to the low abundance of labeled peptides after short pulse times. In addition, detectable labeling can only be achieved in timescale of hours rather than minutes, thus limiting the temporal resolution of p-SILAC for analyses of translation dynamics. This is because pulsed amino acids need to be first taken up by the cells and conjugated to transfer RNA (tRNA), before being incorporated into nascent proteins in detectable quantities, a process that is far from instantaneous. Finally, although SILAC has been utilized *in vivo* through generation of isotopically labeled whole organisms,^42^ p-SILAC has not been extended to *in vivo* analysis yet. Efficient pulse-labeling with SILAC amino acids *in vivo* will be challenging due to the large pool of unlabeled amino acids present. Achieving cell-specificity is also challenging, although it may be possible to employ cell type specific labeling with amino acid precursors (CTAP) to overcome this issue.^43^,^44^ In CTAP, a set of non-native amino acid biosynthesis enzymes are utilized to generate isotope labeled Lys residues from unnatural isotope labeled amino acid precursors. These enzymes can be expressed in a cell-type specific manner, thus enabling cell-specificity in SILAC labeling of Lys residues in co-cultures.^45^ However, it remains to be determined whether CTAP can be combined with pulse-labeling in order to assess translation rates in a cell-specific manner.

In contrast to p-SILAC which uses stable isotopologues, bio-orthogonal non-canonical amino acid tagging (BONCAT)^46^ utilizes pulses of non-canonical amino acids for labeling of nascent proteins (Fig. 2B). Critical to this method is the fact that certain non-canonical amino acids such as azidohomoalanine (Aha), a methionine analogue that contains an active azide moiety, can be incorporated into proteins in the position of their canonical counterpart, due to the permissive nature of certain amino acyl-tRNA synthetases. After incorporation, these amino acids can be either coupled to fluorescent compounds, or to affinity tags such as biotin, *via* Click chemistry.^47^ The former method is known as fluorescent non-canonical amino acid tagging (FUNCAT), and allows overall visualization of nascent proteins *in vitro* and *in vivo*.^48^,^49^ In the latter, biotin labeled nascent proteins are purified from the total protein pool, followed by their identification and quantification *via* LC-MS/MS.^46^ Biotin enables purification under stringent conditions, thus limiting non-specific pulldown of any un-labeled proteins. Crucially, use of BONCAT amino acids does not interfere with synthesis of proteins *in vitro*^46^ and *in vivo*.^49^,^50^ However, the affinities of most cellular amino acyl-tRNA synthetases to non-canonical amino acids are often significantly less than those of their canonical counterpart, which significantly reduces the efficiency of BONCAT labeling in presence of their canonical counterparts. This is a major limiting factor for using BONCAT *in vivo* where cognate canonical amino acid pools cannot be concomitantly removed. Moreover, BONCAT labeling is not cell-type specific on its own, further limiting the usefulness of the method for *in vivo* analysis of translation.

To counter these issues, bacterial amino acyl-tRNA synthetases have been engineered to have a higher affinity for given non-canonical amino acids, and to conjugate such amino acids to specific mammalian tRNAs.^51^ These engineered synthetases can be transgenically expressed in a given cell-type, thus allowing cell-specific BONCAT labeling *in vivo*.^52^,^53^ A related approach named stochastic orthogonal recoding of translation (SORT) relies on genetic code expansion, achieved *via* co-expression of an orthogonal amino acyl-tRNA synthetase/tRNA pair in specific cells. The pyrrolysyl-tRNA synthetase (PylRS)/tRNA-CUA pair is particularly useful, since it can be engineered to accept a wide range of useful unnatural amino acids with diverse chemical side chains. This allows cell-specific incorporation of unnatural amino acids into proteins at specific codons, using various sense codon sequences. SORT has been utilized to specifically label newly synthesized proteins at different tissues and stages of *D. melanogaster* larval development,^54^ and to enrich SORT-tagged proteins for pull-down assays.^55^

Similar to p-SILAC, a major shortcoming of methods which use non-canonical amino acids for analysis of translation, is their low temporal resolution. Another weakness is compromised quantitative accuracy. Pull-down efficiency of nascent proteins is affected by the number and sites of incorporated non-canonical amino acids, as well as the inherent protein-dependent variations in the click chemistry. Moreover, label-free quantification of nascent protein levels across different pull-down samples requires multiple replicates and is only semi-quantitative. These issues are not pertinent to p-SILAC, since labeling efficiency is uniform. In addition, samples that are pulsed with different SILAC amino acids are mixed together when the lysates are generated, which eliminates any variability caused by sample handling, and allows highly accurate relative quantification of labeled peptides within a mixed sample pair. To overcome the quantitative constrains of BONCAT, quantitative non-canonical amino acid tagging (QuaNCAT) combines p-SILAC and BONCAT in the experimental workflow (Fig. 2C).^56^ Briefly, cells are pulsed with both BONCAT and SILAC amino acids. The lysates are then mixed and nascent proteins are purified *via* click chemistry, followed by their identification and quantification by mass spectrometry using the SILAC labeled peptides. QuaNCAT allows accurate relative quantification thanks to the SILAC labels. However, unlike standard p-SILAC, QuaNCAT can achieve a higher proteome coverage due to the enrichment the low-abundant pulse-labeled proteins.^56^ Dual pulsing and enrichment can also improve the temporal resolution, by enabling detection of nascent proteins from shorter pulse times.^57^,^58^ More recently, another method termed Heavy Isotope Labeled Azidohomoalanine Quantification (HILAQ) has been developed, in which light and heavy isotope labeled Aha variants are utilized for both tagging and quantification of nascent proteins.^59^ As opposed to BONCAT and QuaNCAT, in which Click dependent biotin labeling and enrichment is performed at the protein level, in HILAQ the pre-mixed heavy and light Aha pulse-labeled lysates are first trypsin digested, and Click dependent biotin labeling and enrichment is then performed at the peptide level. This allows specific enrichment of Aha containing peptides for mass spectrometry identification and quantification. Thus, while HILAQ is truly quantitative like QuaNCAT, the use of isotope labeled Aha does away with the need for double SILAC-BONCAT labeling and greatly simplifies the experimental workflow.^59^

An alternative method for labeling of nascent proteins is the antibiotic puromycin. Puromycin, produced by the bacterium *Streptomyces*, is an aminonucleoside antibiotic which inhibits translation elongation in both prokaryotic and eukaryotic cells. The broad specificity of puromycin renders it unsuitable for therapeutic use, and it is therefore exclusively used for research purposes. The chemical structure of puromycin shows strong similarity to that of an amino acyl-tRNA molecule. More precisely, puromycin closely resembles the 3′ terminal end of a tyrosyl-tRNA conjugate.^60^,^61^ This attribute gives puromycin the ability to occupy the acceptor site of the ribosome during translation elongation, and become incorporated into the nascent polypeptide chain in a non-selective manner, thanks to the action of ribosome peptidyl-transferase. However, once puromycinylated, the peptidyl chain cannot receive any further amino acids, leading to premature termination of translation, and the eventual falling off of the puromycinylated peptide from the traversing ribosome.

In mammalian cells, puromycin can robustly inhibit translation in the timescale of minutes and at low micro-molar concentrations.^60^,^61^ Importantly, unlike BONCAT or SILAC amino acids, pulsed puromycin is rapidly incorporated into translating poly-peptides, allowing fast labeling of nascent proteins. Such puromycin labeled nascent proteins can be visualized *via* microscopy, or detected by western-blotting or fluorescence-activated cell sorting (FACS), using an anti-puromycin antibody.^62^ Alternatively, Puromycin-associated nascent chain proteomics (PUNCH-P) utilizes a biotinylated variant of puromycin (Biotin-PURO) for global profiling of nascent proteins by LC-MS/MS.^63^ In PUNCH-P, the polysomes are first isolated by sedimentation, followed by labeling of nascent proteins with Biotin-PURO during cell-free translation. Labeled nascent proteins are then purified by streptavidin conjugated beads, and identified by LC-MS/MS, providing an instantaneous snapshot of cellular translation rates at the time of lysis (Fig. 2D). Labeling under cell-free conditions is crucial due to the low cell permeability of Biotin-PURO. This is a major disadvantage for PUNCH-P, as prior lysis and sedimentation of ribosome will result in loss of any spatial regulation that may be influencing the cellular translation rates in live cells. However, since long puromycin incubation times can be performed in cell-free conditions as opposed to live cells, PUNCH-P can achieve a high proteome coverage thanks to accumulating large quantities of puromycylated proteins.^63^

Another variant of puromycin used for global translation monitoring is *O*-propargyl-puromycin (OPP).^64^ OPP contains an alkyne group, which through Click chemistry can be covalently coupled to fluorescent tags for visualization of nascent proteins, or to biotin for their capture and identification by LC-MS/MS.^64^ As opposed to Biotin-PURO, OPP is cell permeable, thus enabling the labeling of nascent proteins to be performed in live cells (Fig. 2D). Moreover, a newly designed OPP analogue which carries a phenylacetyl group (PhAc-OPP), allows labeling to be achieved in a cell-specific manner.^65^ Owing to the added phenylacetyl group, PhAc-OPP is rendered inactive. However, orthogonal expression of the enzyme penicillin G acylase (PGA), which can remove the phenylacetyl group, leads to generation of active OPP in the enzyme expressing cells, thus allowing labeling and subsequent profiling of nascent proteins in a cell-specific manner. Transgenic expression of PGA in specific cell-types of a model organism, could therefore enable cell-specific monitoring of translation rates *in vivo*, using otherwise harmless pulses of PhAc-OPP.

Thanks to the rapid incorporation of puromycin into nascent proteins, puromycin based methods discussed above can at least in theory achieve far better temporal resolutions compared to p-SILAC and BONCAT. However, as mentioned for BONCAT, label-free quantification of nascent protein levels across different captured samples is only semi-quantitative. Although it remains to be demonstrated, combining puromycin based labeling methods with mass spectrometry quantification techniques such SILAC^40^ or tandem mass tagging (TMT),^66^ should overcome this issue in future.

## Live cell imaging of translation

**Fig. 3:**
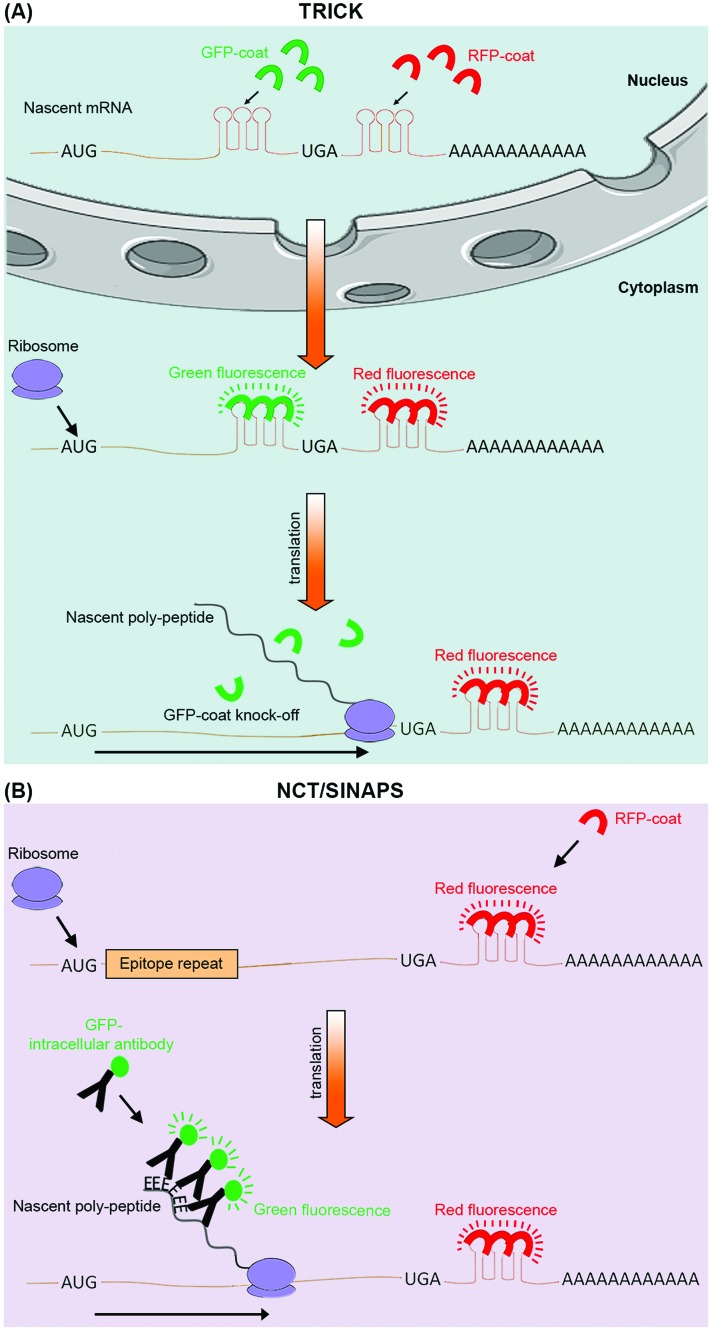
Live cell imaging methods for targeted analysis of translation. (A) TRICK utilizes tagging of a target mRNA by two distinct stretches of hairpin repeats, each of which can be bound by a specific exogenous RBP (coat protein). The first repeat, recognized by a specific RFP tagged coat protein (red), is added after the stop codon (UGA), while the second repeat, recognized by a distinct GFP tagged coat protein (green), is added within the ORF. Before the pioneer round of translation, both types of coat proteins are bound to the mRNA molecule, resulting in it appearing as a yellow spot in the live image feed. However, during the first round of translation, the traversing ribosome displaces the GFP tagged coat proteins, resulting in a change of color from yellow to red in the live image feed. (B) NCT/SINAPS uses concurrent tagging of the target mRNA with a hairpin repeat, along with N-terminal tagging of its coded protein sequence with epitope repeats. Hairpin repeats are added after the stop codon, and recognized by an exogenous RFP tagged coat protein (red), which marks all individual mRNA molecules. As the newly synthesized epitopes (E) exit the ribosomes during translation, they are rapidly recognized by an intracellular GFP labeled antibody (green). This results in the translating mRNA molecule to change appearance from red to yellow in the live image feed. The co-localizing GFP fluorescent intensity can also be used to estimate translation rate dynamics of an individual mRNA molecule. Use of repeats allows fluorescent signal amplification that is needed for single-molecule visualization in both methods.

The next-generation sequencing and proteomics methods discussed above enable global assessment of protein translation rates, but at discrete time points. Thanks to a number of recently developed live cell imaging methodologies, it is now also possible to continuously monitor translation of specific mRNAs in real-time (Table 1). Translating RNA imaging by coat protein knock-off (TRICK) allows live imaging of the first round of translation from single mRNA molecules, in both cultured cells and model organisms.^67^ TRICK utilizes a two-color fluorescent microscopy technique to label a target mRNA after its transcription.^68^ Briefly, a stretch of specific RNA hairpin repeats is added to the 3′UTR of the mRNA. Each hairpin can bind to an exogenous RBP such as the MS2 bacteriophage coat protein (MCP). Expression of a Red Fluorescent Protein (RFP) tagged MCP allows visualization of the target mRNAs in the red fluorescent channel. Importantly, due to the amplification of the fluorescent signal as a result of multiple hairpin repeats, even single mRNA molecules can be detected above the background noise levels. A nuclear localization signal (NLS) is also added to the RFP-MCP in order to enrich it in the nucleus, thus enabling the immediate coating of the target mRNA molecules after transcription. In parallel, a distinct stretch of RNA hairpin repeats, which is similarly recognized by another exogenous RBP, such as the PP7 bacteriophage coat protein (PCP), is added to the same target mRNA but within the ORF region. Expression of a Green Fluorescent Protein (GFP) tagged PCP bearing an NLS allows concomitant detection of the mRNA molecules in the green fluorescent channel. Crucially, prior to the pioneer round of translation, an mRNA molecule is visible in both red and green channels, thus appearing as a yellow spot in the live imaging feed. However, during the first round of translation, the moving ribosome knocks the GFP-PCP molecules off the mRNA ORF region, resulting in loss of green fluorescence, thus the change of color from yellow to red in the live image feed (Fig. 3A). TRICK is a powerful method for visualization of translation initiation at the single molecule level. Nevertheless, it does not inform on the actual rates of protein translation, as it cannot reveal the level of ribosome association with a target mRNA molecule.

Recently, four independent studies reported similar methods for direct visualization of protein translation in live cells, using a combined mRNA and protein fluorescent tagging approach.^69^–^73^ These methods, nicknamed nascent chain tracking (NCT), or single-molecule imaging of nascent peptides (SINAPS), allow continuous monitoring of translation dynamics through visualization of nascent polypeptide chains that are associated with their cognate mRNA molecules.^69^–^73^ In all methods, the target mRNA is tagged and visualized at the single molecule level with a 3′UTR RNA hairpin repeat that is recognized by a fluorescently tagged exogenous RBP. In parallel, the coded protein is N-terminally tagged by multiple epitope repeats which can be recognized at the single molecule level by an intracellular fluorescent antibody. Key to this approach is the use of epitope tags as opposed to direct tagging with conventional fluorescent proteins such as GFP and RFP.^74^ Fluorescent proteins have long maturation times, which means they cannot be utilized for instantaneous visualization of nascent proteins. Use of epitope tags allows nascent proteins to be rapidly detected with pre-expressed intracellular fluorescent antibodies as they exit the translating ribosomes. Co-localization of these detectable nascent chains with their cognate mRNA molecule is indicative of active translation, and their fluorescence intensity acts as an indicator of the amount of translation that is occurring from a single mRNA molecule, at a given time (Fig. 3B).^69^–^73^

Although not a global approach, NCT/SINAPS is an extremely powerful methodology for targeted live cell monitoring of translation initiation, elongation, and termination, over long periods of time (>1 h).^69^–^73^ But perhaps the greatest advantage of NCT/SINAPS is its ability to assess the spatiotemporal dynamics of translation at the single molecule level, thus revealing the intra-cellular as well as inter-cellular heterogeneity in translational control, a feat that is not possible *via* next-generation sequencing or proteomics based global methods which report population averaged translation rates. Crucially, although NCT/SINAPS has not been adapted for *in vivo* use yet, this should be possible in the near future through genome editing of endogenous alleles in a cell specific manner, thus enabling live spatiotemporal assessment of translation dynamics in more physiological contexts.

## Discussion

Compared to our substantial understanding of transcriptional networks and their regulation, knowledge of translational regulation in different biological processes is still in its infancy. Thanks to several newly developed methods, however, this imbalance is beginning to be redressed. As summarized in this review, these methods employ diverse technologies from next-generation sequencing and proteomics to single-molecule live cell fluorescent imaging, in order to enable researchers to map and measure protein translation, *in vitro* and *in vivo*. As these methods become more readily available to different researchers, it is likely that multiple approaches will be used in combination for a more comprehensive assessment of translational regulation. For instance, ribo-seq can be used as a pioneering experiment, defining the translatome of a given cell. This can then be followed by proteomics approaches such as p-SILAC or BONCAT for rapid assessment of changes in translation rates of different ORFs, across multiple cell states or treatments, using a tailored reference protein sequence database derived from the ribo-seq data, for all subsequent proteomics searches.^75^–^77^

Crucially, despite the here discussed recent advances in monitoring protein translation, several technological hurdles still remain to be overcome. For instance, ribosome profiling studies have revealed a significant degree of translation occurring outside of the canonical ORFs. However, detection of these non-canonical, often short, ORFs at the protein level have been extremely challenging due to their low abundance.^78^ Further advances in mass spectrometry, namely more sensitive and faster instrumentations, are required to enable detection of these non-canonical ORFs at the protein level, paving the way for better assessment of their cellular functions. Moreover, a wealth of recent evidence indicates that many cellular mRNAs can undergo diverse RNA base modifications.^79^ Many such modifications may affect protein translation, but this remains to be systematically demonstrated. Novel methods for global translation monitoring that are integrated with approaches which globally assess RNA modifications are needed in order to systematically interrogate the impact of different base modifications on translational regulation. Finally, while translation is often dysregulated in many pathological conditions such as neuronal disorders and cancers, the methodologies for assessment of protein translation have not been yet extended to clinical settings. It remains to be determined whether the available approaches can move beyond cells and model organisms and be adapted for assessment of translational dysregulation in clinical settings.

## Glossary

Amino acyl-tRNA synthetase: a family of enzymes which catalyze the covalent conjugation of specific amino acids to their corresponding tRNA molecules.

Azidohomoalanine (Aha): an amino acid analogue of methionine which contains an azido moiety.

Bio-orthogonal non-canonical amino acid tagging (BONCAT): a method for monitoring translation through pulse-labeling with non-canonical amino acids, followed by their affinity capture and LC-MS/MS analysis.

Biotin acceptor peptide (Avi Tag): a 15 amino acid peptide sequence which can be recognized and biotinylated by the biotin ligase BirA.

Biotin ligase (BirA): a biotin ligase enzyme, originally from *E. coli*, which is used to catalyze biotinylation of Avi tags.

Cell type specific labeling with amino acid precursors (CTAP): a method for cell-specific SILAC labeling of cells by non-mammalian amino acid precursor-processing enzymes to convert isotope labeled lysine precursors into labeled lysine in a given cell.

Chromatin immunoprecipitation (ChIP): an affinity purification method for purification of DNA segments attached to a specific protein.

ChIP followed by micro-array chip hybridization (ChIP-chip): a method for systematic identification of DNA segments bound by a specific protein through micro-array analysis of ChIP samples.

ChIP followed by next-generation sequencing (ChIP-seq): a method for systematic identification of DNA segments bound by a specific protein through next-generation sequencing analysis of ChIP samples.

Click chemistry: a simple set of high yielding reactions based on azido and alkyne moieties that allow covalent conjugation of two or more molecules in a stereospecific manner.

DNA micro-array chip hybridization (micro-array): a method for assessment of gene expression based on hybridization to a collection of microscopic DNA spots attached to a solid surface.

Fluorescence-activated cell sorting (FACS): a flow cytometry method for sorting a heterogeneous mixture of fluorescently labeled cells, one cell at a time, based on their specific fluorescent properties.

Fluorescent non-canonical amino acid tagging (FUNCAT): a method for visualization of translation through pulse-labeling of nascent proteins with non-canonical amino acids, followed by their conjugation to fluorescent tags for microscopy analysis.

Green fluorescent protein (GFP): a fluorescent protein derived from the jelly fish *Aequorea victoria* which exhibits green fluorescence when exposed to light in the blue to ultraviolet range.

Heavy isotope labeled azidohomoalanine quantification (HILAQ): a modified version of BONCAT for quantitative monitoring translation, which utilizes pulse-labeling with isotope labeled Aha, followed by trypsin digestion, affinity capture of Aha containing peptides, and their relative quantification *via* LC-MS/MS analysis.

Liquid chromatography coupled with tandem mass spectrometry (LC-MS/MS): an analytical chemistry method which combines liquid chromatography with online tandem mass spectrometry, in order to resolve and analyze complex sample mixtures.

Messenger RNA (mRNA): a family of cellular RNA which convey genetic information from DNA to the ribosomes for protein synthesis.

MS2 bacteriophage coat protein (MCP): an RNA binding protein from the MS2 bacteriophage, which is an icosahedral, single-stranded RNA virus that infects *E. coli*.

Nascent chain tracking (NCT): a method for live cell imaging of translation which utilizes concurrent single molecule visualization of nascent proteins and their cognate mRNA.

Nuclear localization signal (NLS): a stretch of positively charged amino acids that mark proteins for import into the nucleus by the nuclear transport machinery.

Open reading frame (ORF): a stretch of codon sequences in an mRNA which do not contain a stop codon.

*O*-Propargyl-puromycin (OPP): a cell permeable variant of puromycin containing an alkyne group.

Polysome profiling: a global method for monitoring protein translation *via* micro-array or RNA-seq based quantification of polysome associated mRNAs.

PP7 bacteriophage coat protein (PCP): an RNA binding protein from the PP7 bacteriophage, which is an icosahedral, single-stranded RNA virus that infects *Pseudomonas aeruginosa*.

Proximity-specific ribosome profiling: a variant of ribo-seq which uses proximity-specific biotinylation for tagging and separation of local ribosome associated mRNAs.

Pulsed-SILAC (p-SILAC): a method for monitoring translation through pulse-labeling the cells with SILAC amino acids, followed by LC-MS/MS analysis.

Puromycin-associated nascent chain proteomics (PUNCH-P): a method for monitoring translation through pulse-labeling translating ribosomes with Biotin-PURO in cell-free conditions, followed by the capture and analysis of labeled nascent proteins *via* LC-MS/MS.

Quantitative non-canonical amino acid tagging (QuaNCAT): a method that combines p-SILAC and BONCAT through concurrent pulse-labeling with SILAC and BONCAT amino acids, followed by their affinity capture and quantification *via* LC-MS/MS.

Red fluorescent protein (RFP): an engineered monomeric red fluorescent protein derived from the *Discosoma sp.* fluorescent protein ‘DsRed’.

Ribonucleoprotein (RNP): a protein–RNA complex.

Ribosome profiling (ribo-seq): a next-generation sequencing based method for assessment of translation through separation of translating ribosomes and sequencing of mRNA ribosome footprints.

RNA binding protein (RBP): a protein which can bind to double or single stranded RNA molecules.

RNA-sequencing (RNA-seq): a next-generation sequencing method for assessment of the sequence and quantity of RNA molecules in a biological sample.

Stable isotope labeling of amino acids in culture (SILAC): a mass spectrometry based method for quantifying differences in protein abundance among different samples *via* non-radioactive isotopically labeled amino acids.

Short ORFs (sORF): an ORF which is smaller than 300 nucleotide (100 amino acids).

Single-molecule imaging of nascent peptides (SINAPS): a method for live cell imaging of translation which utilizes concurrent single molecule visualization of nascent proteins and their cognate mRNA.

Stochastic orthogonal recoding of translation (SORT): a method for cell-specific monitoring of translation through ectopic expression of an orthogonal amino acyl-tRNA synthetase/tRNA pair, allowing cell-specific pulse-labeling with non-canonical amino acids at diverse sense codons.

Transfer RNA (tRNA): a type of cellular RNA which functions by bringing amino acids to the ribosome during protein synthesis.

Translatome: collection of all proteins formed by translation of cellular mRNAs.

Translating ribosome affinity purification-sequencing (TRAP-seq): a variant of ribo-seq which uses Immunoprecipitation of tagged ribosomal proteins for separation of ribosome associated mRNAs.

Translating RNA imaging by coat protein knock-off (TRICK): a single molecule two-color fluorescent live cell imaging method, in which mRNAs are constitutively detected in one fluorescent channel, but detection in the second channel is abrogated upon the first round of translation.

Upstream ORFs (uORF): a small ORF which is situated within the 5′UTR of an mRNA.

Un-translated region (UTR): a section of mRNA that is immediately situated upstream (5′) or downstream (3′) of an ORF.

## Conflicts of interest

There are no conflicts to declare.
